# Soft Ferromagnetic Bulk Metallic Glass with Potential Self-Healing Ability

**DOI:** 10.3390/ma13061319

**Published:** 2020-03-14

**Authors:** Parthiban Ramasamy, Mihai Stoica, Gabriel Ababei, Nicoleta Lupu, Jürgen Eckert

**Affiliations:** 1 Erich Schmid Institute of Materials Science, Austrian Academy of Sciences, Jahnstraße 12, Leoben A-8700, Austria; 2 Laboratory of Metal Physics and Technology, Department of Materials, ETH Zurich, 8093 Zurich, Switzerland; 3 National Institute of Research and Development for Technical Physics, 47 Mangeron Boulevard, RO-700050 Iaşi, Romania; 4 Department of Materials Science, Chair of Materials Physics, Montanuniversität Leoben, Jahnstraße 12, Leoben A-8700, Austria

**Keywords:** bulk metallic glasses, mechanical properties, thermal stability, doping, self-healing

## Abstract

A new concept of soft ferromagnetic bulk metallic glass (BMG) with self-healing ability is proposed. The specific [Fe_36_Co_36_B_19.2_Si_4.8_Nb_4_]_100−x_(Ga)_x_ (x = 0, 0.5, 1 and1.5) BMGs prepared by copper mold casting were investigated as a function of Ga content. The Ga-containing BMGs still hold soft magnetic properties and exhibit large plastic strain of 1.53% in compression. Local melting during shearing produces molten droplets of several µm size throughout the fracture surface. This concept of local melting during shearing can be utilized to produce BMGs with self-healing ability. The molten regions play a vital role in deflecting shear transformation zones, thereby enhancing the mechanical properties.

## 1. Introduction

Among several bulk metallic glass (BMGs) systems, Fe-based BMGs are considered as a class of engineering materials with enormous potential for many fields of application due to their unique combination of excellent magnetic properties, good corrosion resistance, high hardness, high fracture strength, and low cost [1,2,3]. However, these Fe-based BMGs do not exhibit strain hardening and cannot sustain plastic deformation at room temperature. This brittle behavior under mechanical loading severely limits their application as engineering and structural materials. In recent years, huge efforts have been undertaken to improve the plastic deformability of monolithic BMGs or BMG composites [4]. Several researchers have proved that plastic deformability in metallic glasses can be largely enhanced by introducing different microstructural heterogeneities such as nano-crystals, phase separation, or short-/medium-ranged clusters into the glassy matrix by controlling the casting process [5,6,7,8]. In the case of Fe-based BMGs, the room temperature plasticity was improved by the addition of small amounts of soft elements like Ni, Er, and Cu [9,10,11,12]. In recent years, among the several Fe-based glassy alloys Fe-Co-B-Si-Nb BMGs have been regarded as one of the most promising alloys because of its combined advantages of functional and structural materials [1].

Motivated by these results, we considered to dope a Fe_36_Co_36_B_19.2_Si_4.8_Nb_4_ base alloy (hereafter named FeCoBSiNb) with Ga. Since the compositional variation of the Ga content in steps of 0.1 at.% is very difficult, we considered adding Ga in steps of 0.5 at.%. In previous works, Ga was employed as a main alloying element to improve the glass-forming ability (GFA) in a few glass-forming alloy systems [13,14,15]. However, in the present study, Ga is considered as a doping element to improve the plastic deformability of Fe-based BMGs without hindering their GFA. 

## 2. Materials and Methods

In this work, Ga-containing glassy samples were produced in 3 steps. An eutectic Fe25Nb75 (wt.%) pre-alloy, together with the crystalline B (99 wt.%), crystalline Si (99.99 wt.%), Fe and Co lumps (99.9 wt.%), were melted together by induction heating under a protective Argon atmosphere. To this master alloy the desired amount of Ga (99.99 wt.%) was added through arc-melting. Cylindrical samples were prepared by remelting the pieces of the master alloys in quartz tubes and subsequently injecting into a water-cooled copper mold. Structural characterization of the as-cast and annealed samples was done by X-ray diffraction (XRD) using a PHILIPS PW 1050 diffractometer with Co-K_α_ radiation (λ = 0.17889 nm). Microstructural investigation of the as-cast glassy samples was performed using a Carl Zeiss Libra 200MC High Resolution Transmission Microscope (HRTEM). The thermal behavior of the glassy samples was evaluated with a differential scanning calorimeter (DSC) NETZSCH DSC 404 C with an accuracy of ± 2 K. To study the structural evaluation, samples were annealed for 5 min. at different temperatures using the same DSC, with a constant heating and cooling rate of 20 K/min. For compression testing, an INSTRON 8562 device was used in constant strain rate mode at room temperature. The fracture surfaces of the samples were investigated using a scanning electron microscopy (SEM) Zeiss LEO 1525, equipped with energy-dispersive X-ray spectroscopy (EDS). For magnetic measurements, DC M-H hysteresis loops were recorded with a vibrating sample magnetometer (VSM) at ambient temperature.

## 3. Results and Discussion 

### 3.1. Structural and Thermal Characterization

**Figure 1:**
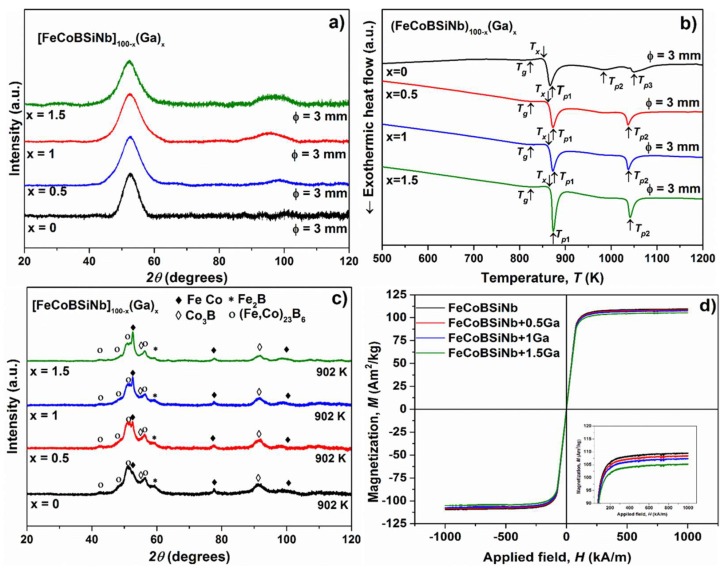
(**a**) XRD patterns and (**b**) DSC traces (heating rate 20 K/min) of as-cast [FeCoBSiNb]_100−x_(Ga)_x_ (x = 0, 0.5, 1 and 1.5) glassy samples with maximum achievable diameter for each composition; (**c**) XRD patterns of [FeCoBSiNb]_100−x_ (Ga)_x_ (x = 0, 0.5, 1 and 1.5) samples heated up to the end of the first crystallization event; (**d**) Hysteresis loops for 1 mm diameter as-cast [FeCoBSiNb]_100−x_(Ga)_x_ (x = 0, 0.5, 1 and 1.5) glassy samples. The inset shows the behavior of the Ga-doped samples close to the saturation region, i.e., between 80 and 100 Am^2^/kg.

Figure 1a shows the XRD pattern of as-cast (FeCoBSiNb)_100−x_(Ga)_x_ (x = 0, 0.5, 1, 1.5) glassy samples with maximum achievable diameter for each composition. 

All samples exhibit similar diffuse patterns without any distinct crystal diffraction peaks indicating the formation of a glassy structure. However, a second broad diffraction maximum is observed around 98.35° for the samples containing 1 and 1.5 at.% Ga. According to Bernal’s dense random packing hard sphere model [16], the position of the first and second broad diffraction maxima contain information about mean interatomic distances and medium-range order (MRO) in glassy systems. Therefore, it can be assumed that the substitution of Ga atoms leads to some atomic level rearrangements. As reported in the literature, the critical diameter (*D_c_*) for this FeCoBSiNb alloy is 5 mm [1]. If the alloy is fluxed with B_2_O_3_ prior to casting then *D_c_* can be increased up to 7.7 mm [17]. In our work, following the above described experimental route, *D_c_* is 3 mm. The difference in *D_c_* could be due to the purity of the starting raw materials, the casting technique and the casting parameters used. The addition of Ga does not affect the GFA of this FeCoBSiNb alloy and *D_c_* (3 mm) also remains the same up to 1.5 at.% Ga addition. This may be due to the relatively large atomic radius of Ga (1.53 nm) and negative heats of mixing for Fe-Ga, Co-Ga pairs [18]. 

The DSC traces of as-cast glassy samples with *D_c_* are shown in Figure 1b. For all compositions, the DSC traces display a clear glass transition event, followed by a supercooled liquid region (SLR) and crystallization, further confirming the glassy structure of these alloys. The thermal properties, such as onset temperature of crystallization *T_x_*, glass transition temperature *T_g_*, crystallization peak temperatures (*T_p_*_1_, *T_p_*_2_ and *T_p_*_3_), liquidus temperature *T_liq_*, extension of the SLR *ΔT_x_* (*ΔT_x_* = *T_x_* − *T_g_*), the GFA parameter *γ* (*γ* = *T_x_*/(*T_g_* + *T_liq_*)), and the reduced glass transition temperature *T_rg_* (*T_rg_* = *T_g_*/*T_liq_*) for the present BMGs are listed in Table 1. The addition of Ga does not affect *T_g_*, but *T_x_* marginally increases from 858 K to 863 K for both 0.5 and 1 at.% Ga, and to 869 K for 1.5 at.% Ga-added glasses. On the other hand, the two small exothermic events *T_p_*_2_ and *T_p_*_3_ observed for FeCoBSiNb glass disappears. Instead, a single strong exothermic event *T_p_*_2_ is observed for Ga-added glasses.

Figure 1c illustrates the XRD patterns of the (FeCoBSiNb)_100−x_Ga_x_ (x = 0, 0.5, 1and 1.5) glassy samples after constant-rate heating in the DSC up to the end of their first crystallization event, which is at 902 K. The crystallization sequence of the BMGs containing 0 and 0.5 at.% Ga is almost identical. The BMGs with 1 and 1.5 at.% Ga show distinct bcc-(Fe,Co) peaks along with (Fe,Co)_23_B_6_-type peaks at the end of the first crystallization event. The formation of the bcc-(Fe,Co) phase for BMGs with higher Ga contents may be associated to the presence of Ga atoms in the matrix, which may aid the nucleation of the bcc-(Fe,Co) phase. The absence of bcc-(Fe,Co) phase for lower Ga contents indicates that 0.5 at.% of Ga is not sufficient to trigger the nucleation of this phase.

The M-H hysteresis loops of the different BMGs are shown in Figure 1d. From the hysteresis loops it is clear that saturation occurs at relatively similar magnetic fields for all glassy samples. The saturation magnetization (*M_s_*) decreases marginally from 108 to 105 Am^2^/kg when the Ga content increases from 0 to 1.5 at.%. This decrease in *M_s_* is attributed to the addition of the non-magnetic element Ga.

**Figure 2:**
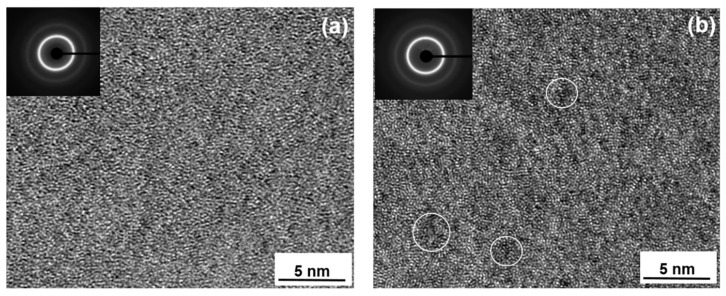
Bright-field HRTEM images of the as-cast (**a**) FeCoBSiNb and (**b**) [FeCoBSiNb]_99.5_Ga_0.5_ glasses. The insets show the corresponding fast Fourier transformed (FFT) images. Some features of local ordering/clusters are observed, which are marked by the white circles.

High resolution transmission electron microscopy (HRTEM) investigations were carried out to understand the effect of Ga addition in FeCoBSiNb glass. As-cast FeCoBSiNb and FeCoBSiNb + 0.5Ga samples were subjected to wide-angle X-ray diffraction before preparing for HRTEM investigations and the samples were amorphous. HRTEM micrographs for FeCoBSiNb and FeCoBSiNb+0.5Ga samples are shown in Figure 2a,b, together with their corresponding fast Fourier transformed (FFT) images (shown as insets). The as-cast FeCoBSiNb + 0.5Ga glass exhibits some locally ordered regions, which are marked by white circles in the image, but it is not evident whether the Ga atoms cause this ordering/cluster structure. The exact identification of the chemical composition of these locally ordered regions is not easy due to their very small size and the fact that they are dispersed in a chemically complex multicomponent glassy matrix. EDX analysis indicates a faint clustering of Ga atoms. 

However, it should be noted that the presence of ordered zones/clusters in a glassy matrix does not necessarily imply that they act as nucleation sites for crystal growth. The ordered embryos must be larger than the critical nucleus size in order for their growth to be thermodynamically favorable [19,20,21]. Since no ordered regions were observed in the Ga- free glass, it can be assumed that these ordered zones are due to the Ga atoms. From the above structural evaluation and thermal stability data it is evident that these ordered zones do not act as heterogeneous nucleation sites, thereby the GFA remains unaffected.

### 3.2. Mechanical Properties 

**Figure 3:**
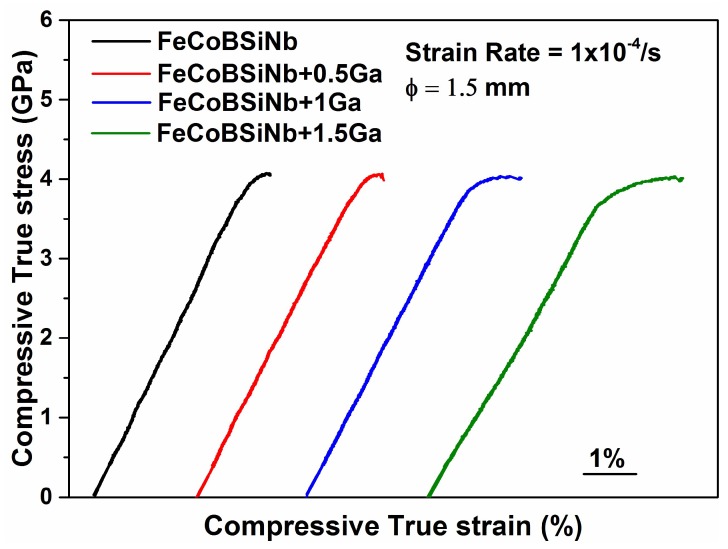
Room temperature compressive true stress-true strain curves for (FeCoBSiNb)_100−x_(Ga_x_) (x = 0, 0.5, 1, and 1.5) glasses measured at a strain rate of 1 × 10^−4^/s.

The room temperature compressive true stress-true strain curves for the as-cast [FeCoBSiNb]_100−x_(Ga_x_)(x = 0, 0.5, 1 and 1.5) BMGs are shown in Figure 3. All the curves are offset from the origin for better visibility. All samples exhibit an elastic deformation regime followed by a small compressive plastic regime. From the stress-strain curves it is evident that the plastic strain increases gradually with increasing Ga content. 

For the 0.5 at.% Ga-added sample, the yield strength *σ_y_*, elastic strain *ɛ_y_*, fracture strength *σ_f_* and plastic strain *ɛ_pl_* are almost the same as for the FeCoBSiNb parent glass, i.e., 3.95 ± 0.02 GPa, 2.80%, 4.06 ± 0.02 GPa and 0.43%, respectively. For higher amounts of Ga addition, the plastic deformation is improved. The yield stress is 3.71and 3.62 ± 0.2 GPa for the 1 and 1.5 at.% Ga-added samples and their corresponding elastic strain values are 2.73% and 3.30%, respectively. The fracture of all Ga-added samples occurs at nearly the same value of true stress, around 4 GPa, but the corresponding fracture strain is different: 3.87% for 1 at.% Ga- and 4.83% for 1.5 at.% Ga-added samples. The plastic deformation of the samples extends up to 1.07% and 1.53% for 1 at.% and 1.5 at.% Ga-added samples. The improvement in the plastic deformability of the glassy rods containing Ga can be explained based on the ordered zones observed in the TEM investigations. The dispersed ordered zones seem to be responsible for promoting plastic deformability in the Ga-containing samples, which is not observed for the Ga-free samples loaded under the same conditions. The ordered zones present in the Ga-containing samples affect the localized deformation in the glassy matrix. They may hinder shear band propagation by acting as pinning centers or by deflecting the shear bands into multiple small branches. The volume and density of these ordered zones also play a crucial role in deflecting the shear bands. If they are distributed densely in a narrow zone, then the deflection will be very less leading to brittle failure. The plasticity increase in the stress-strain curves reveals that the volume fraction and density of these ordered zones are large enough for affecting the shear band propagation through the material but not too high to cause distinct ductile failure. Sarac et al. [22] in their recent work showed that the presence of soft zones/nanocrystals in an amorphous matrix plays a key role in improving the plasticity by controlling the shear propagation rate.

### 3.3. Fracture Surface Analysis

The fracture surface morphology of the samples after the compression tests was investigated using SEM. The fracture surface of the FeCoBSiNb and FeCoBSiNb + 0.5Ga samples consists of a number of small fracture zones and their zone planes appear to be inclined by about 70°–90° to the direction of the applied load. This kind of failure is mainly due to the simultaneous generation of a large number of small facture events at many sites, at a very high stress level close to 4.0 GPa. The high fracture strength of these glasses is mainly due to the covalent bonding nature of their constituents [1,23]. Although a slight increase in plastic strain is observed for FeCoBSiNb + 0.5Ga samples, the overall appearance of the fracture surface points towards a brittle fracture mode, with many shells indicating the generation and propagation of cracks. Neither shear bands on the lateral surfaces nor typical vein patterns are observed on the fracture surface of these samples.

**Figure 4:**
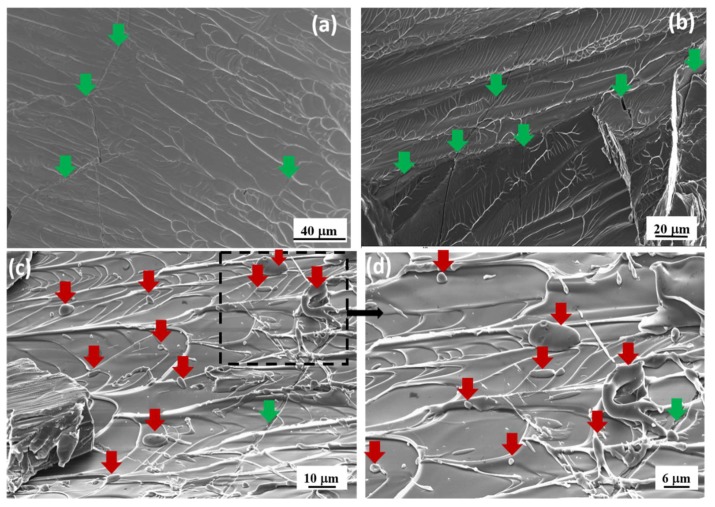
(**a**) and (**b**) SEM micrographs of a (FeCoBSiNb)_99_Ga_1_ sample, showing the fracture surface after the compression test at different regions with different magnifications. Cracks are marked by green arrows; (**c**) (FeCoBSiNb)_98.5_Ga_1.5_ sample, fracture surface at the center of the sample after the compression test and (**d**) center region at higher magnification (square region in (**c**)). Droplets are marked by red arrows and cracks are marked by green arrows.

More interesting features are observed for the samples containing 1 and 1.5 at.% Ga. Figure 4a,b show the fracture surface of the FeCoBSiNb + 1Ga samples after compression testing taken from different regions with different magnifications. The fracture surfaces contain a large number of vein patterns together with several micro-cracks (indicated by green arrows in the image). At first the material starts to deform plastically; after a while brittle fracture starts, because the cracks are superimposed on the vein patterns, indicating a combination of ductile and brittle failure modes.

Figure 4c represents the fracture surface of the FeCoBSiNb + 1.5Ga samples taken from the center of the samples after compression testing and Figure 4d shows a magnified image of the area marked with dotted lines in Figure 4c. The red arrows indicate metal droplets, which are observed throughout the fracture surface. Compared to the 1 at.% Ga samples, the fracture surface of the 1.5 at.% Ga samples displays more vein patterns and less micro-cracks. Again, the micro-cracks are superimposed on the vein patterns. The presence of metal droplets on the fracture surfaces suggests that at the very high stress level of 4.0 GPa, the flowed layers must have melted, as is commonly observed in case of deformable BMGs [24]. In a very recent work Stoica et al. [11] showed that during compression of FeCoBSiNb + 0.5Cu glass the temperature in the shear plane can raise up to 1352 K, by assuming a maximum shear propagation rate, i.e., 0.9Vs (Vs = speed of sound). The raise in temperature (1352 K) is just 30 K above the solidus temperature of the FeCoBSiNb + 0.5Cu alloy and barely enough to melt very thin layers. The solidus temperatures of FeCoBSiNb + 0.5Cu and (FeCoBSiNb]_100−x_(Ga)_x_ (x = 0, 0.5, 1 and 1.5) alloys are ≈1310 K [25] (supplementary Figure S1). In case of FeCoBSiNb + 0.5Cu glass [11] no such molten droplets were found on the fracture surface but for FeCoBSiNb + 1.5Ga glass µm-sized molten droplets are observed throughout the fracture surface (see Figure 4c,d. EDS analysis performed on the molten droplets indicates a minor increase in Ga content compared to the other fractured regions without any melting (supplementary Figure S2 and Table S1). Researchers have already proved that Ga has a tendency to form nano-precipitates with Fe [26,27,28]. From these results it can be assumed that the ordered zones formed due to the addition of Ga atoms play an important role in deflecting shear bands and its contributing to some local melting due to the rise in temperature during shear band propagation. EBSD measurements were carried out to identify the nature of the molten droplets. The absence of Kikuchi bands indicates that they are amorphous (supplementary Figure S3). This proves that the cooling rate is high enough to freeze the molten region instantly.

## 4. Conclusions

In summary, we have revealed that Ga can be considered as a potential doping element for Fe_36_Co_36_B_19.2_Si_4.8_Nb_4_ glass; it improves glass-forming ability as well as plastic deformability to about 1.07% and 1.53% plastic strain for 1 and 1.5 at.% Ga addition without deteriorating the magnetic properties. Furthermore, for the first time we reported the presence of molten regions of several µm size throughout the facture surface in Fe-based BMG. Local melting at crack tips accompanied by fast cooling can be considered as a promising healing mechanism for improving industrial applications of Fe-based BMGs in many ways.

## Figures and Tables

**Table 1:** Thermal stability parameters *T_g_*, *T_x_*, *T_p_*_1_, *T_p_*_2_, *T_p_*_3_, *T_liq_*, *T_rg_*, *γ* and *ΔT_x_* as a function of Ga content for [FeCoBSiNb]_100-x_(Ga)_x_ (x =0, 0.5, 1 and 1.5) BMGs.

Compositions	*T_g_* (K)	*T_x_* (K)	*T_p_*_1_ (K)	*T_p_*_2_ (K)	*T_p_*_3_ (K)	*T_liq_* (K)	*T_rg_*	*γ*	*ΔTx* (K)
FeCoBSiNb	825	858	867	985	1050	1475	0.56	0.373	33
FeCoBSiNb + 0.5Ga	825	863	873	1037	-	1475	0.56	0.375	38
FeCoBSiNb + 1Ga	825	863	873	1038	-	1440	0.57	0.381	38
FeCoBSiNb + 1.5Ga	825	869	873	1038	-	1440	0.57	0.383	41

## Supplementary Materials

The following are available online at https://www.mdpi.com/1996-1944/13/6/1319/s1, Figure S1: DSC traces (heating rate 20 K/min) of as-cast [FeCoBSiNb]_100−x_(Ga)_x_ (x = 0, 0.5, 1 and 1.5) glassy samples, Figure S2. SEM, EDS and EBSD results obtained on a [FeCoBSiNb]_98.5_Ga_1.5_ sample after the compression test; Figure S3. (a), (b), (c) and (d) are the EBSD patterns obtained from the fracture surface for the points 1, 2, 3 and 4, respectively, Table S1. EDS results of all the points in Atomic percent (%). Spectra 1, 2 and 3 were recorded on molten region, spectra 4 and 5 are on fractured zone without melting.

## References

[1] Inoue A., Shen B., Chang C. (2004). Super-high strength of over 4000 MPa for Fe-based bulk glassy alloys in [(Fe_1−x_Co_x_)_0.75_B_0.2_Si_0.05_]_96_Nb_4_ system. Acta Mater..

[2] Inoue A., Shinohara Y., Gook J.S. (1995). Thermal and Magnetic Properties of Bulk Fe-Based Glassy Alloys Prepared by Copper Mold Casting. Mater. Trans. JIM.

[3] Stoica M., Eckert J., Roth S., Zhang Z.F., Schultz L., Wang W.H. (2005). Mechanical behavior of Fe_65.5_Cr_4_Mo_4_Ga_4_P_12_C_5_B_5.5_ bulk metallic glass. Intermetallics.

[4] Trexler M.M., Thadhani N.N. (2010). Mechanical properties of bulk metallic glasses. Prog. Mater. Sci..

[5] Hui X., Liu S., Pang S., Zhuo L., Zhang T., Chen G., Liu Z. (2010). High-zirconium-based bulk metallic glasses with large plasticity. Scripta Mater..

[6] Calin M., Eckert J., Schultz L. (2003). Improved mechanical behavior of Cu–Ti-based bulk metallic glass by in situ formation of nanoscale precipitates. Scripta Mater..

[7] Fan C., Inoue A. (2000). Ductility of bulk nanocrystalline composites and metallic glasses at room temperature. Appl. Phys. Lett..

[8] Zhang L., Cheng Y.-Q., Cao A.-J., Xu J., Ma E. (2009). Bulk metallic glasses with large plasticity: Composition design from the structural perspective. Acta Mater..

[9] Inoue A., Shen B.L. (2004). A New Fe-based Bulk Glassy Alloy with Outstanding Mechanical Properties. Adv. Mater..

[10] Gu X., McDermott A., Poon S.J., Shiflet G.J. (2006). Critical Poisson’s ratio for plasticity in Fe–Mo–C–B–Ln bulk amorphous steel. Appl. Phys. Lett..

[11] Stoica M., Scudino S., Bednarčik J., Kaban I., Eckert J. (2014). FeCoSiBNbCu bulk metallic glass with large compressive deformability studied by time-resolved synchrotron X-ray diffraction. J. Appl. Phys..

[12] Jiao Z., Li H., Gao J., Wu Y., Lu Z. (2011). Effects of alloying elements on glass formation, mechanical and soft-magnetic properties of Fe-based metallic glasses. Intermetallics.

[13] Gan Z., Yi H., Pu J., Wang J., Xiao J. (2003). Preparation of bulk amorphous Fe–Ni–P–B–Ga alloys from industrial raw materials. Scripta Mater..

[14] Pawlik P., Davies H., Gibbs M. (2004). The glass forming abilities and magnetic properties of Fe–Al–Ga–P–B–Si and Fe–Al–Ga–P–B–C alloys. Mater. Sci. Eng. A.

[15] Shen B.-L., Koshiba H., Mizushima T., Inoue A. (2000). Bulk amorphous Fe–Ga–P–B–C alloys with a large supercooled liquid region. Mater. Trans. JIM.

[16] Bernal J. (1960). Geometry of the structure of monatomic liquids. Nature.

[17] Bitoh T., Makino A., Inoue A., Greer A. (2006). Large bulk soft magnetic [(Fe_0.5_Co_0.5_)_0.75_B_0.20_Si_0.05_]_96_Nb_4_ glassy alloy prepared by B_2_O_3_ flux melting and water quenching. Appl. Phys. Lett..

[18] Takeuchi A., Inoue A. (2005). Classification of bulk metallic glasses by atomic size difference, heat of mixing and period of constituent elements and its application to characterization of the main alloying element. Mater. Trans..

[19] Turnbull D. (1952). Kinetics of solidification of supercooled liquid mercury droplets. J. Chem. Phys..

[20] Herlach D.M. (1994). Non-equilibrium solidification of undercooled metallic metls. Mater. Sci. Eng. R.

[21] Christian J.W. (2002). CHAPTER 10—The Classical Theory of Nucleation. The Theory of Transformations in Metals and Alloys.

[22] Sarac B., Ivanov Y.P., Chuvilin A., Schöberl T., Stoica M., Zhang Z., Eckert J. (2018). Origin of large plasticity and multiscale effects in iron-based metallic glasses. Nat. Commun..

[23] Inoue A. (2000). Stabilization of metallic supercooled liquid and bulk amorphous alloys. Acta Mater..

[24] Greer A., Cheng Y., Ma E. (2013). Shear bands in metallic glasses. Mater. Sci. Eng. R.

[25] Parthiban R., Stoica M., Kaban I., Ravi K., Eckert J. (2015). Viscosity and fragility of the supercooled liquids and melts from the Fe–Co–B–Si–Nb and Fe–Mo–P–C–B–Si glass-forming alloy systems. Intermetallics.

[26] Bhattacharyya S., Jinschek J.R., Li J.F., Viehland D. (2010). Nanoscale precipitates in magnetostrictive Fe_1−x_Ga_x_ alloys for 0.1 <x <0.23. J. Alloys Compd..

[27] Ikeda O., Kainuma R., Ohnuma I., Fukamichi K., Ishida K. (2002). Phase equilibria and stability of ordered b.c.c. phases in the Fe-rich portion of the Fe–Ga system. J. Alloys Compd..

[28] Petculescu G., Wu R., McQueeney R., Buschow K.H.J. (2012). Chapter three—Magnetoelasticity of bcc Fe–Ga Alloys. Handbook of Magnetic Materials.

