# Supplementary material for: Soft Ferromagnetic Bulk Metallic Glass with Potential Self-Healing Ability
Source: Materials (Basel). 2020 Mar 14;13(6):1319. doi: 10.3390/ma13061319 (PMC7143147; doi:10.3390/ma13061319)
Supplement: Supplementary file 1 [file materials-13-01319-s001.pdf]

*materials*

MDPI

Supplementary Materials

# Soft Ferromagnetic Bulk Metallic Glass with Potential Self-Healing Ability

**Parthiban Ramasamy [1,*], Mihai Stoica [2], Gabriel Ababei [3], Nicoleta Lupu [3] and Jürgen Eckert [1,4]**

1 Erich Schmid Institute of Materials Science, Austrian Academy of Sciences, Jahnstraße 12, A 8700 Leoben, Austria; juergen.eckert@oeaw.ac.at
2 Laboratory of Metal Physics and Technology, Department of Materials, ETH Zurich, 8093 Zurich, Switzerland; mihai.stoica@mat.ethz.ch
3 National Institute of Research and Development for Technical Physics, 47 Mangeron Boulevard, Iaşi, RO-700050, Romania; gababei@phys-iasi.ro (G.A.); nicole@phys-iasi.ro (N.L.)
4 Department of Materials Science, Chair of Materials Physics, Montanuniversität Leoben, Jahnstraße 12, A-8700 Leoben, Austria
* Correspondence:Correspondence: parthiban.ramasamy@oeaw.ac.at

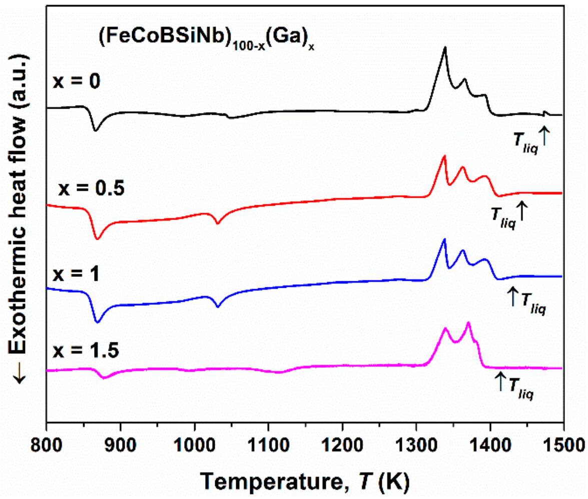


**Figure S1.** DSC traces (heating rate 20 K/min) of as-cast $[FeCoBSiNb]_{100-x}(Ga)_x$ (x =0, 0.5, 1 and 1.5) glassy samples.

Figure 1 depicts the DSC traces of as-cast glassy samples up to 1500 K. The liquidus temperatures of the $[FeCoBSiNb]_{100-x}(Ga)_x$ (x =0, 0.5, 1 and 1.5) glasses are 1475 ± 5, 1475 ± 5, 1440 ± 5 and 1440 ± 5 K, respectively.

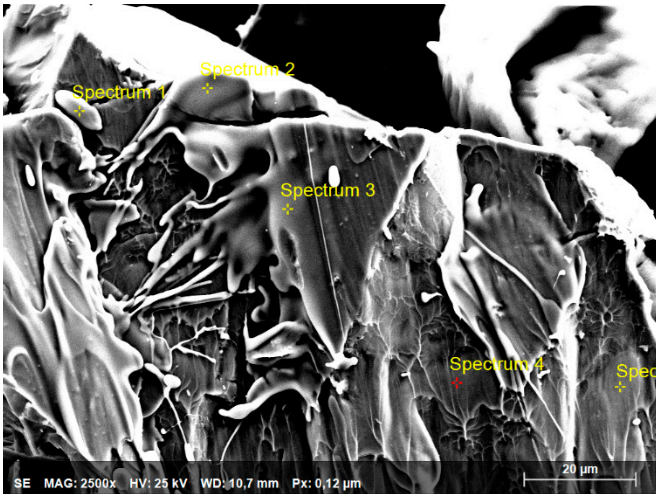

**Figure S2.** SEM, EDS and EBSD results obtained on a $[FeCoBSiNb]_{98.5}Ga_{1.5}$ sample after the compression test.

Figure 2 shows a fracture surface image of the $[FeCoBSiNb]_{98.5}Ga_{1.5}$ amorphous rods used for the mechanical testing. The chemical composition (in at.%) obtained at different regions using EDS are given in Table 1. The EDS results prove the existence of Ga-rich and Ga-poor regions in the sample. The spectra 1, 2 and 3 were recorded on molten regions, whereas 4 and 5 are on fractured zones without melting.

**Table S1.** EDS results of all the points in Atomic percent (%). Spectra 1, 2 and 3 were recorded on molten region, spectra 4 and 5 are on fractured zone without melting.

| Spectrum | Carbon | Oxygen | Silicon | Iron | Cobalt | Gallium | Niobium |
|---|---|---|---|---|---|---|---|
| 1 | 0,00 | 0,00 | 6,00 | 43,16 | 43,64 | 1,73 | 5,46 |
| 2 | 0,00 | 0,00 | 6,29 | 43,15 | 43,27 | 1,60 | 5,69 |
| 3 | 0,00 | 0,00 | 6,19 | 43,10 | 43,48 | 1,80 | 5,37 |
| 4 | 0,00 | 0,00 | 5,81 | 43,67 | 43,91 | 1,25 | 5,41 |
| 5 | 0,00 | 0,00 | 5,28 | 43,04 | 45,13 | 1,53 | 5,02 |
| Mean | 0,00 | 0,00 | 6,09 | 43,14 | 43,61 | 1,60 | 5,54 |
| Sigma | 0,00 | 0,00 | 0,19 | 0,09 | 0,24 | 0,02 | 0,11 |
| Sigma mean | | | 0,08 | 0,04 | 0,11 | 0,0, | 0,05 |

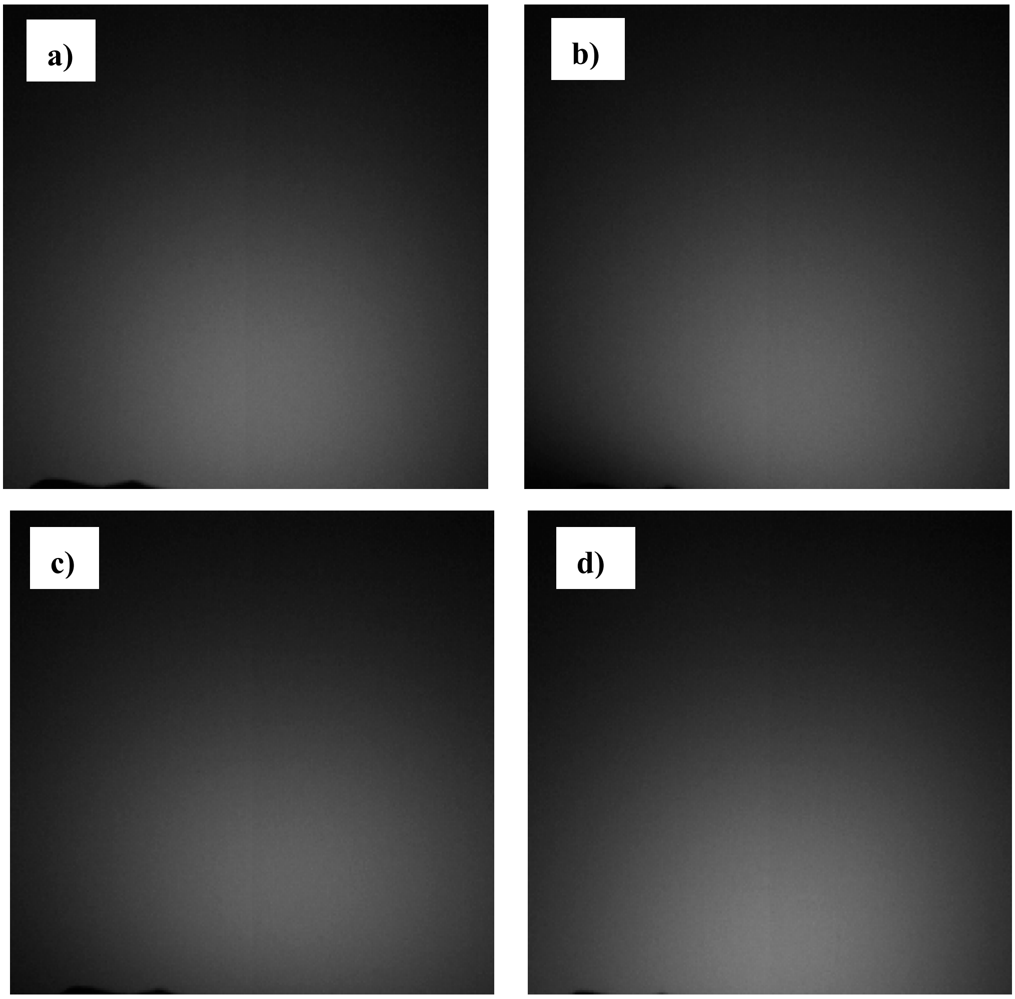


**Figure S3.** (**a**), (**b**), (**c**) and (**d**) are the EBSD patterns obtained from the fracture surface for the points 1, 2, 3 and 4, respectively.

Figure 3 shows the EBSD patterns obtained for the same spots where EDS measurements were done. The spectra 1, 2 and 3 are on molten regions, whereas 4 is on fractured zone without melting. The absence of Kikuchi bands indicates the amorphous nature of the molten droplets.
